# The Stabilization of S100A9 Structure by Calcium Inhibits the Formation of Amyloid Fibrils

**DOI:** 10.3390/ijms241713200

**Published:** 2023-08-25

**Authors:** Ella Sanders, Rebecca Csondor, Darius Šulskis, Ieva Baronaitė, Vytautas Smirnovas, Luckshi Maheswaran, Jack Horrocks, Rory Munro, Christina Georgiadou, Istvan Horvath, Ludmilla A. Morozova-Roche, Philip T. F. Williamson

**Affiliations:** 1 Centre for Biological Sciences, University of Southampton, Southampton SO17 1BJ, UK; 2 Sector of Amyloid Research, Institute of Biotechnology, Life Sciences Centre, Vilnius University, LT-10257 Vilnius, Lithuania; 3 Department of Medical Biochemistry and Biophysics, Umeå University, SE-90187 Umeå, Sweden

**Keywords:** amyloid, S100A9, protein stability, neurodegenerative disease, Alzheimer’s disease, Parkinson’s disease

## Abstract

The calcium-binding protein S100A9 is recognized as an important component of the brain neuroinflammatory response to the onset and development of neurodegenerative disease. S100A9 is intrinsically amyloidogenic and in vivo co-aggregates with amyloid-β peptide and α-synuclein in Alzheimer’s and Parkinson’s diseases, respectively. It is widely accepted that calcium dyshomeostasis plays an important role in the onset and development of these diseases, and studies have shown that elevated levels of calcium limit the potential for S100A9 to adopt a fibrillar structure. The exact mechanism by which calcium exerts its influence on the aggregation process remains unclear. Here we demonstrate that despite S100A9 exhibiting α-helical secondary structure in the absence of calcium, the protein exhibits significant plasticity with interconversion between different conformational states occurring on the micro- to milli-second timescale. This plasticity allows the population of conformational states that favour the onset of fibril formation. Magic-angle spinning solid-state NMR studies of the resulting S100A9 fibrils reveal that the S100A9 adopts a single structurally well-defined rigid fibrillar core surrounded by a shell of approximately 15–20 mobile residues, a structure that persists even when fibrils are produced in the presence of calcium ions. These studies highlight how the dysregulation of metal ion concentrations can influence the conformational equilibria of this important neuroinflammatory protein to influence the rate and nature of the amyloid deposits formed.

## 1. Introduction

The classical hallmarks of Alzheimer’s (AD) and Parkinson’s Diseases (PD) are the formation of amyloid deposits within the brain; however, patients also exhibit chronic inflammation, and there is growing evidence that this plays a role in the onset and progression of these diseases [1,2]. Patients typically exhibit elevated levels of proinflammatory markers [3,4], including the intrinsically amyloidogenic calcium-binding protein S100A9, which is heavily expressed at sites of inflammation within the brain [4,5,6]. S100A9’s role in neurodegenerative disease is the subject of much investigation, as studies of S100A9 gene knockdowns in mice AD models have revealed a significant reduction in amyloid load and improved cognitive function [7]. S100A9 amyloids were found to colocalise with amyloid-β peptide plaques in patients with AD and mild-cognitive impairment [4,8], with α-synuclein in Lewy bodies in patients with Parkinson’s disease [5], and form precursor plaques and amyloid aggregates in the cells and tissues after traumatic brain injury as a precursor state for AD [9]. These findings are mirrored in in vitro studies revealing that the presence of S100A9 can significantly reduce the lag phase and modulate the rates of growth of both amyloid-β peptide and α-synuclein fibrils [10,11]. These properties reflect the potential for S100A9 to template itself onto the surface of amyloid-β peptide(1–42) fibrils, blocking secondary nucleation pathways and potential reducing the overall amyloid load [12]. Given the association of S100A9 with these amyloid deposits and the influence it has on aggregation of the major polypeptides involved in AD and PD, a clearer understanding of how it exerts its action will clearly be of benefit in our understanding of disease progression. A clearer understanding of S100A9’s own aggregation potential will allow us to begin to determine how S100A9 acts on these other proteins, whether it exhibits its action against the soluble species, later oligomeric intermediates or the fibrillar state [13].

Calcium dyshomeostasis is known to play an important role in AD and PD, with variations in both intra- and extracellular concentration contributing to the onset and progression of AD and PD [14,15,16,17,18]. As a member of the S100 family of Ca^2+^-binding proteins, these fluctuations in Ca^2+^ concentration would be expected to influence the regulatory activity of both the intra- and extra-cellular populations of S100A9 [18,19,20,21]. This family of proteins possesses two helix-loop-helix EF hand Ca^2+^-binding domains with a high-affinity site at the C-terminus and a lower affinity site at the N-terminus [22]. These two domains are linked by a more flexible hydrophobic linker region [22]. In vivo, S100A9 is typically found as a heterodimer with its homologous protein S100A8, an interaction that is promoted by elevated Ca^2+^ levels [21,23]. In response to inflammatory challenges, S100A9 is also excreted from the cell as a stable homodimer whose structure and stability is influenced by the presence of Ca^2+^ [24,25]. S100A9 also exhibits affinity for other divalent metal ions, including Mn^2+^, Zn^2+^ and Cu^2+^, a property that plays an important role in this protein’s antimicrobial activity [26] and in the regulation of metal ion homeostasis within the brain [5,25].

Given the propensity of S100A9 to interact with this broad range of metal ions, it is not unexpected that these can influence the rate of S100A9 fibril formation [27], and indeed, studies have revealed that elevated levels of Ca^2+^ can significantly attenuate the rate at which fibrilization occurs, presumably through the stabilization of the protein structure as differential scanning calorimetry studies, showing higher melting temperatures for S100A9 in the presence of Ca^2+^ [23]. Despite these studies, our understanding of the conformational transitions that result in the conversion of S100A9 from its native to its fibrillar form, and the influence of Ca^2+^ ions on the resulting structures remain poorly understood. Given the role that S100A9 plays in modulating the deposition of both amyloid-β peptide and α-synuclein, it is important to understand how Ca^2+^ influences the conformational equilibria of S100A9 and how this impacts both S100A9 aggregation and its interactions with other amyloidogenic proteins [10,11]. In this study, we have used a combination of synchrotron radiation circular dichroism in conjunction with solution-state NMR to demonstrate that the presence of Ca^2+^ inhibits slow fluctuations in S100A9 structure that appear necessary for the onset of nucleation and subsequent fibril formation. Analysis of the S100A9 fibrils by magic-angle spinning solid-state NMR revealed that S100A9 fibrils grown in the absence of Ca^2+^ ions were structural homogeneous, with 85–90% of the residues within S100A9 adopting a rigid core with the remaining residues forming a more mobile region surrounding Tyr_20_. A comparison of the spectra obtained from S100A9 fibrils grown in the presence of non-limiting concentrations of Ca^2+^ ions exhibited similar solid-state NMR spectra, indicating that despite the ability of Ca^2+^ to modulate the plasticity of the soluble S100A9, it exerts no effect on the resulting fibrillar structure.

## 2. Results and Discussion

### 2.1. Influence of Ca^2+^ on the Rate of S100A9 Fibril Formation

**Figure 1:**
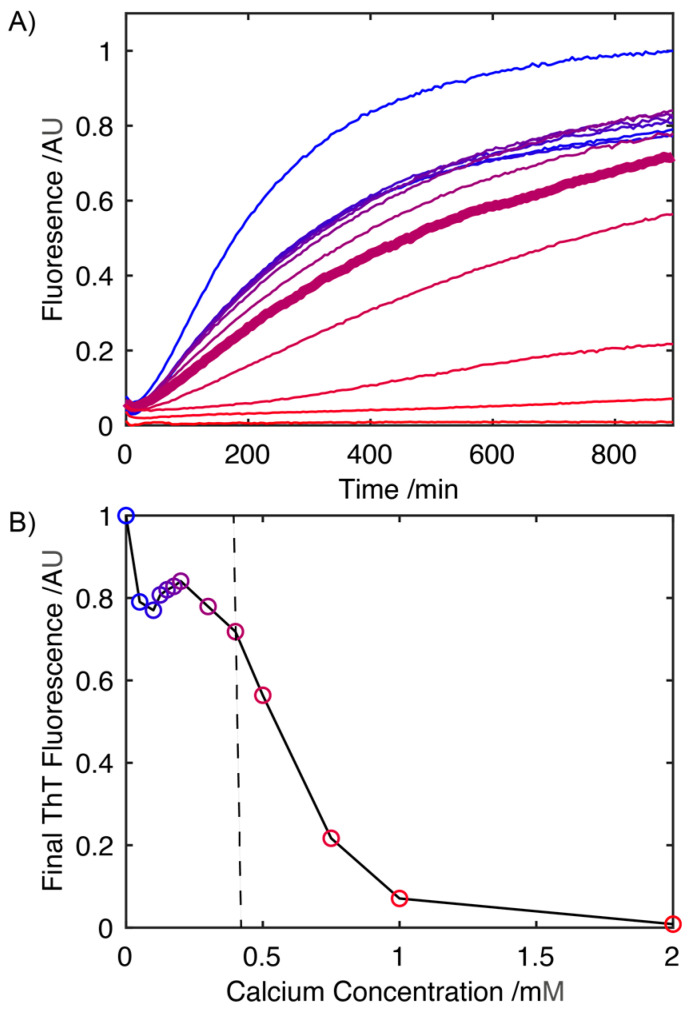
Influence of Ca^2+^ on the kinetics of S100A9 (3.3 mg mL^−1^) Thioflavin-T positive fibril formation in 20 mM Tris buffer (pH 7.2) (**A**). Fibril formation was monitored at 0, 0.05, 0.10, 0.125, 0.15, 0.175, 0.20, 0.30,0.40, 0.50, 0.75, 1.00 and 2.00 mM concentrations of Ca^2+^ ions (concentration of Ca^2+^ ions increasing from blue to red, with colouring matching the data points in (**B**)). The concentration at which S100A9 aggregation starts to be suppressed significantly by Ca^2+^, 0.4 mM is highlighted in bold (**A**). Corresponding plot of Thioflavin-T fluorescence at the plateau level after S100A9 incubated at 41 °C for 15 h, highlighting the drop in total amount of S100A9 fibrils formed as the Ca^2+^ concentration is increased (**B**). Vertical line (in (**B**)) represents the concentration at which the Ca^2+^ ions begin to supress the S100A9 aggregation (data point corresponds to the trace in bold in (**A**)).

The rate of fibril formation was monitored using a Thioflavin-T (ThT) fluorescence assay, where the fluorescence at 485 nm is proportional to the extent of conversion of S100A9 into its fibrillar form. In the absence of Ca^2+^, fibril formation occurs at 41 °C in the absence of any mechanical agitation and begins to plateau after 15 h (Figure 1A). Increasing the concentration of CaCl_2_ results in a progressive drop in the rate at which S100A9 is converted to its fibrillar form, which is reflected in decreasing slope of the fibrillation curves (Figure 1A). An analysis of the ThT fluorescence after 15 h (Figure 1B) reveals that at concentrations of Ca^2+^ ions up to ~400 mM, there is little change in the overall level at which the ThT fluorescence plateaus, indicating that at these concentrations, Ca^2+^ has little impact on the extent of the conversion of S100A9 into its fibrillar form. Above 400 mM there is a significant drop in the level at which the fluorescence intensity plateaus, indicative of a reduction in the total amount of S100A9 that is converted into its fibrillar state. Above 1 mM Ca^2+^, the absence of any increase in the ThT fluorescence indicates that S100A9 fibril formation is largely suppressed.

### 2.2. Influence of Ca^2+^ on the Structure of S100A9

**Figure 2:**
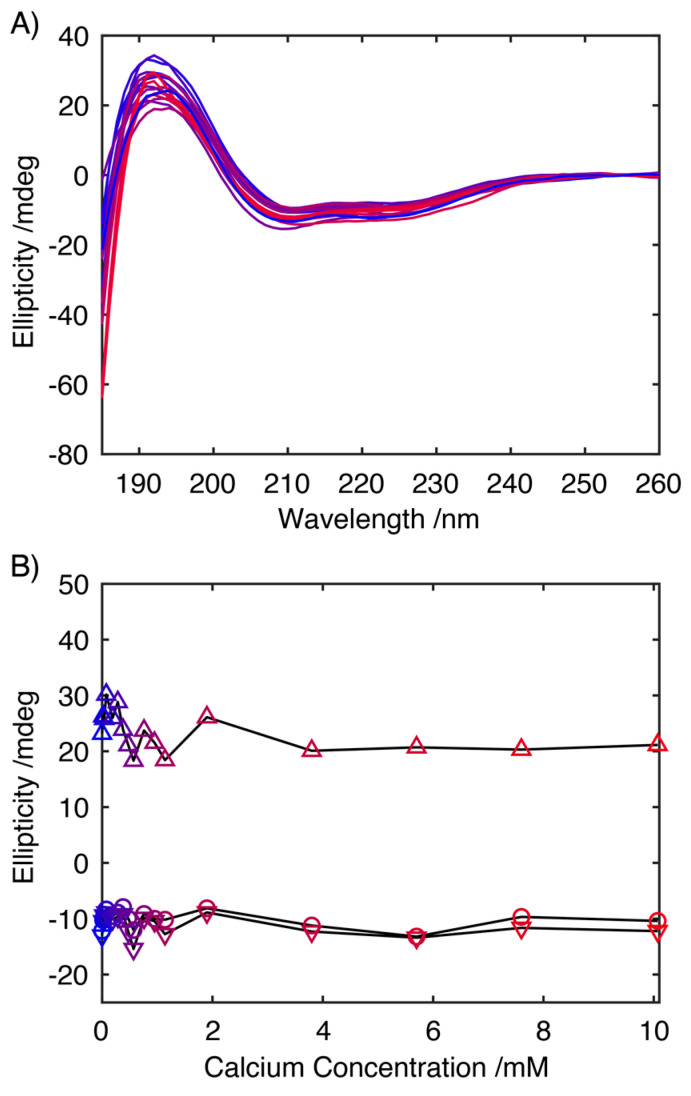
Far-UV CD spectra of S100A9 at 0.5 mg mL^−1^ in 20 mM Tris buffer (pH 7.2) with CaCl_2_ at 0.0, 0.038, 0.076, 0.019, 0.285, 0.380, 0.570, 0.850, 1.900, 3.800, 5.700 and 10.070 mM (increasing concentration of Ca^2+^ turning from blue to red, with colouring matching the data points in (**B**)) (**A**). Plot of the ellipticity at 195 (△), 208 (▽) and 222 nm (◯), the main CD transitions associated with an α-helical conformation, highlighting the absence of any major change in helical structure in response to varying concentrations of Ca^2+^ ions (**B**).

To assess the influence of Ca^2+^ on the secondary structure of S100A9 in solution, synchrotron radiation CD spectra were recorded as this facilitated the measurement of wavelengths as low in the far UV as 185 nm to be analysed even in the presence of increasing CaCl_2_ concentrations, allowing a more rigorous analysis of the secondary structure (Figure 2) [28]. Qualitative analysis of the CD spectrum in the absence of Ca^2+^, indicates a predominantly helical conformation, with characteristic minima at 208 and 222 nm and a maximum at ~195 nm. Increasing the concentration of Ca^2+^ up to 10 mM had little effect on the CD spectra (Figure 2A), with spectra retaining features characteristic of an α-helical structure, with the intensities of the transition associated with a α-helical secondary structure, showing no systematic variation in intensity (Figure 2B). A quantitative analysis of the secondary structure using the SELCON algorithm and basis set 7 revealed that in the absence of Ca^2+^, S100A9 was composed of 51% α-helical structures, 4% β-stranded structures and with 44% present as turns or disordered structure. This distribution of secondary structures mirrors that observed for the published NMR structure of S100A9, which was obtained in 50 mM Tris, 100 mM NaCl, 2 mM CaCl_2_, where the protein was 63% α-helical, 6% β-stranded and with 31% of the sequence either in turns or disordered [29]. A similar analysis of the data acquired at higher Ca^2+^ concentrations showed no systematic variation in the secondary structural with average contributions of 44.9 ± 2.9% from α-helical structures, 16.9 ± 7.1% from β-stranded structures and the remaining 38.7 ± 5.1%, arising from turns and disordered structures. The absence of any significant changes in the CD spectra in response to the increasing Ca^2+^ concentration indicates that the large change in the propensity for S100A9 to form fibrils does not arise from large scale changes in secondary structure induced through the interaction of S100A9 with Ca^2+^.

**Figure 3:**
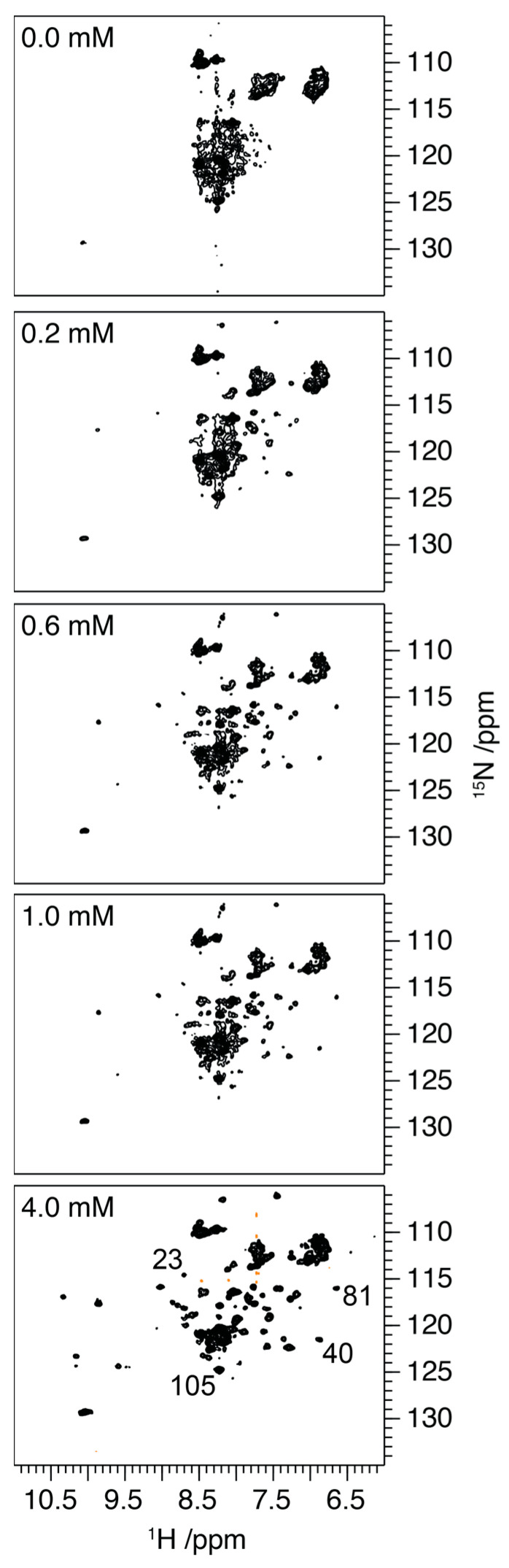
^1^H/^15^N HSQC spectra of S100A9 (100 mM in 20 mM bisTris buffer, pH 7.2) with 0.0, 0.2, 0.6, 1.0 and 4.0 mM CaCl_2_. The distribution of resonances in a ^1^H/^15^N HSQC spectrum provides a fingerprint of the conformational state of the protein, with a greater dispersion of the resonances reflecting a more structured protein (for full details, see main text). Numbering of lower panel shows representative assignments. For full assignment, see Supplementary Figure S1.

To corroborate these observations, ^1^H/^15^N HSQC spectra of ^15^N-labelled S100A9 were recorded as the spectra reflect the structure that the protein adopts in the solution (Figure 3). In the absence of Ca^2+^ and in the presence of 1 mM EDTA, the spread of proton resonances within the HSQC is approximately 0.8 ppm, whilst the ^15^N chemical shifts are clustered at ~110 ppm, ~117 ppm and ~120–127 ppm. Under these conditions, the limited chemical shift dispersion and line broadening make a detailed assignment impossible; however, the resonances are loosely clustered in the ^15^N dimension according to the class of amino acid to which they belong [30]. This apparent conflict between the well-defined structural elements reported by CD, and NMR spectra consistent with less folded structures has previously been reported for molten globule intermediates on a protein folding pathway [31]. These intermediates exhibit well-defined secondary structural elements, but they retain significant conformational plasticity, sampling a range of conformational states on the micro- to milli-second timescale. Although this permits the observation of well-defined secondary structures on the timescale of the CD experiment, the dynamics on the micro- to millisecond timescale results in significant broadening of the NMR resonances [31].

Titration of the S100A9 with increasing concentrations of Ca^2+^ ions results in an increased chemical shift dispersion in both the ^1^H and ^15^N dimensions, indicative of a more well-defined and less conformationally flexible structure. The significant change in spectral dispersion arises between 0.4 and 0.6 mM of Ca^2+^ ions with further increases in dispersion as concentrations are increased from 1.0 to 4.0 mM. We attribute the increased chemical shift dispersion to the binding of Ca^2+^ to the two EF hands, and a reduction in the conformational plasticity, that is observed on the micro- to milli-second timescale in the absence of Ca^2+^. Comparison of the experimental ^1H^/^15^N HSQC with published assignments of S100A9 in the presence of Ca^2+^ reveals a similar chemical shift distribution indicating that the protein adopts a similar conformational state here to that published (Supplementary Figure S1) [29]. The C-terminal EF hand has been observed to exhibit a higher affinity for Ca^2+^ than the N-terminal domain [32,33], being saturated, and potentially more structured, at lower Ca^2+^ concentrations. Interestingly though the increase in chemical shift dispersion arising upon the addition of Ca^2+^ occurs through the ordering of the entire protein backbone, with representative resonances appearing from both the N and C terminal EF hands at Ca^2+^ concentrations as low as 0.4 mM (Figure 3 and Supplementary Figure S1). This suggests that the reduction in conformational plasticity occurs throughout the protein and does not arise through the sequential folding of specific motifs as may have been expected.

**Figure 4:**
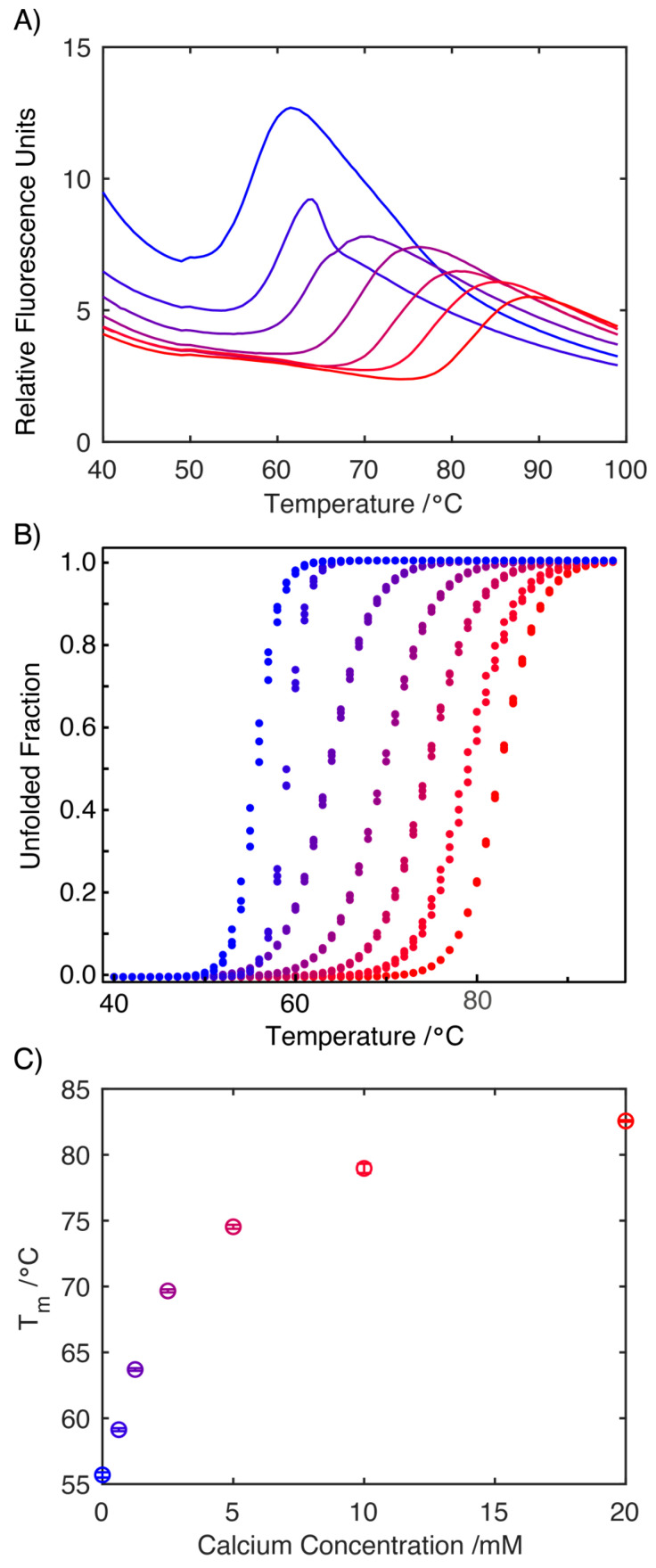
Differential scanning fluorimetry traces of S100A9 (1.3 mg mL^−1^ with 100 mM DNS in 20 mM Tris Buffer, pH 7.2) in the presence of increasing concentrations of Ca^2+^ ions. Measurements were conducted at Ca^2+^ concentrations of 0.0, 0.625, 1.25, 2.5, 5.0, 10.0 and 20.0 mM (increasing from blue to red, colors retained in panels (**B**,**C**)) (**A**). The two-state equilibrium model, MoltenProt, was applied to plot the normalized unfolding transition curves and extract T_m_ (**B**). Plot of the change in T_m_ (°C) in response to increasing concentration of Ca^2+^ ions highlighting the stabilizing effect Ca^2+^ ions have on the structure of S100A9 (**C**).

To assess if the decreased conformational flexibility observed by NMR was reflected in the stability of the protein, a differential scanning fluorimetry study was performed (Figure 4A). Fitting the data to a two-state folding model allowed the melting temperature, T_m_, of the S100A9 to be determined under increasing concentrations of Ca^2+^ ions (Figure 4B). This analysis reveals that increasing Ca^2+^ concentrations result in a significant increase in the melting temperature, T_m_, reflecting the increased stability of the protein. This observation mirrors earlier differential scanning calorimetry experiments, which also reported a significant increase in the melting temperature, T_m,_ upon the binding of Ca^2+^ to S100A9 [23]. Importantly, the cooperative two-state unfolding transition was observed also in the apo-form of S100A9, which corroborates the CD finding that the protein is structured but this structure is less stable in the absence of calcium.

Collectively these results highlight that in the apo-form of S100A9, whilst exhibiting well-defined secondary structural elements as ascertained by CD spectroscopy, the protein retains significant conformational plasticity with extensive dynamics on the micro- to milli-second timescale. The addition of Ca^2+^ appears to suppress this conformational flexibility and increase the stability of the protein. These observations mirror those reported by hydrogen–deuterium exchange mass spectrometry and molecular dynamics studies [25]. Importantly, this loss in structural plasticity is correlated with a reduction in the potential for S100A9 to form a fibrillar structure as determined by Thioflavin-T fluorescence assays. This suggests that conformational flexibility on the micro-/milli-second timescale facilitates the populations of states with a propensity to form amyloid fibrils and a reduction in this conformational flexibility is sufficient to suppress the onset of fibril formation.

The implication of these observations for S100A9 fibrillization in vivo is likely to reflect its localizations. Within the cell Ca^2+^, ion concentrations are typically maintained at levels close to 100 nM, with most Ca^2+^ ions complexed to Ca^2+^-binding proteins. In response to signalling events, the intracellular concentrations may exhibit transient 100-fold increases in Ca^2+^ concentration. Our observations indicate that S100A9 under such conditions would be pre-disposed to form fibrillar-like aggregates. The extracellular concentration of Ca^2+^ ions within the brain is reported as 1.2 mM, based on the concentrations in the cerebrospinal fluid; however, the complex architecture within the brain provides opportunities for local fluctuations with local concentration in the interstitial fluid potentially controlled by astrocyte uptake [34]. Under the millimolar concentrations, that are required for normal neuronal function though, the extracellular pools of S100A9 are likely to remain as a stable well-structured soluble assembly. This variation in the potential for S100A9 to form fibrillar structures, depending on its localization, suggests its mode of action on amyloid-β peptide, which is deposited extracellularly, and α-synculein, that is deposited extracellularly, may be different. However, calcium dyshomeostasis is known to play an important role in both AD and PD, with variations in both intra- and extra-cellular concentrations arising through miss-regulation of Ca^2+^ transport [14,15,16,17,18] as well as disruption of membrane integrity. The implications for this on the aggregation of S100A9, will depend critically on the localization of the S100A9 and the temporospatial distribution of the Ca^2+^ ions within the brain. However, we have demonstrated that over the range of Ca^2+^ ion concentrations reported in vivo, significant variations in the rates of S100A9 aggregation would be observed. Furthermore, any mis-regulation of Ca^2+^ ion concentrations is likely to occur over a range that influences the rate of S100A9 deposition with potential downstream impacts on the deposition of proteins such as amyloid-β peptide or α-synuclein.

### 2.3. Does the Presence of Ca^2+^ Influence the Structure of Fibrillar S100A9?

Our studies of S100A9 in solution indicate that the presence of Ca^2+^ increase structural stability and decrease conformational plasticity on micro- to milli-second time scale, minimising the propensity for S100A9 to aggregate. At low concentrations, variations are observed in the ^1^H/^15^N HSQC spectra, that indicate that S100A9 is still undergoing extensive conformational exchange (Figure 3). To assess whether these changes in conformational plasticity, brought about through the binding of non-inhibitory concentration of Ca^2+^ to S100A9, influenced the structure adopted by S100A9 within the fibrils, magic-angle spinning (MAS) solid-state NMR studies were conducted on fibrils grown in 0.0 and 0.4 mM CaCl_2_.

**Figure 5:**
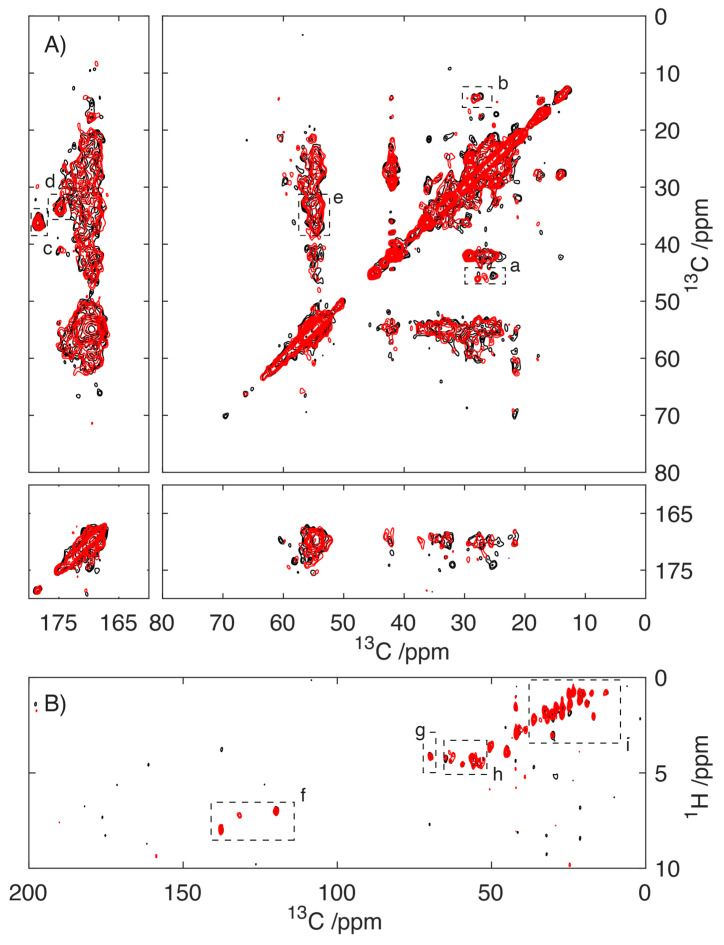
Solid-state NMR studies of S100A9 fibrils prepared in the absence (black) and presence (red) of 0.4 mM CaCl_2_. Cross-polarization 2D ^13^C/^13^C-MAS-DAR spectrum (75 ms DAR mixing) of S100A9 fibrils detecting the rigid core of the S100A9 fibrils (**A**). Highlighted regions with Leu (a), Ile (b), Glu (c), Gln (d) and Glu/Gln (e). Two-dimensional ^1^H/^13^C-MAS-rf-INEPT experiments of S100A9 fibrils detecting the mobile regions within the amyloid fibrils (**B**). Highlighted regions of Tyr (f), Thr (g), C_a_/H_a_ (h), aliphatics (i).

Spectra of S100A9 fibrils prepared in the presence of 0.4 mM CaCl_2_ were compared with those prepared in a Ca^2+^ free environment by using magic-angle spinning NMR studies. Two-dimensional ^13^C/^13^C cross-polarization dipolar-assisted rotational resonance (DARR)-MAS spectra utilize the strong dipolar couplings that are present in the solid-state to enhance the sensitivity and report on the proximity of ^13^C sites with respect to one another [35,36]. As these dipolar couplings are readily averaged by molecular motion, the resonances apparent in the spectra of S100A9 (Figure 5A) arise primarily from the rigid core of the amyloid fibril. In contrast, the ^1^H/^13^C refocussed-INEPT experiment relies on the scalar J-couplings to enhance the signal and establish connectivity (Figure 5B) [37]. INEPT experiments are typically inefficient in the solid-state; however, in the presence of molecular motion, the J-couplings, which are largely unaffected by molecular motion, allow the selective detection of the mobile regions of the amyloid fibrils [38]. Comparison of the spectra of S100A9 fibrils grown in 0.4 mM Ca^2+^ and Ca^2+^ free environment reveals no major differences. The distribution of resonances within both the DARR spectrum and the INEPT spectrum reflects the structures that S100A9 adopts within its fibrillar state. The absence of significant differences indicates that the presence of Ca^2+^ during the aggregation process does not influence the structure(s) that S100A9 adopts in its fibrillar state.

The data presented do not permit accurate analysis of the S100A9 structure, as a complete assignment of the resonances is lacking. An analysis of the ^13^C/^13^C correlation spectrum (Figure 5) reveals that despite spectra crowding, resonances within each envelop are well defined with line widths between 0.5 and 0.75 ppm, consistent with the S100A9 adopting a well-defined structure. This indicates within the sample that S100A9 has adopted a single well-defined conformation. The distribution of resonances within the spectra were compared to shifts in the known structure using the PLUQ software [39]. Although a detailed assignment is precluded, it is possible to assign envelopes of resonances that can be assigned to Leu, Ile, Glu and Gln (Figure 5A). These spectral envelopes are located in regions of the spectra typically associated with these amino acids adopting a β-stranded conformation, as it would be expected that S100A9 adopted the cross-β structure expected in its fibrillar state. This represents a significant change in conformation given the α-helical conformation of the S100A9 in its soluble, native state. This is highlighted in the comparison of the 2D-^13^C/^13^C-DAR spectrum of microcrystalline S100A9 with respect to the fibrillar form (Supplementary Figure S2).

The structural homogeneity is also reflected in the rf-INEPT data (Figure 5B), which reports on the more mobile regions of the amyloid fibrils, where approximately 12 resonances can be identified in the region, that we can tentatively assign to resonances arising from H_a_/C_a_ correlations centred around 50–55 ppm (Figure 5B, h). Due to the characteristic chemical shifts in the Tyr sidechain and the presence of only one such residue in S100A9, it is possible to unambiguously assign the sidechain of Tyr20 (Figure 5B, f), which located this residue in a more mobile region of the protein. Similarly, resonances arising at approximately 70 ppm are typical of threonine sidechains (Figure 5B, g) suggesting the presence of a threonine residue in the mobile region. The presence of additional resonances in the up-field part of the spectrum is consistent with the mobile domain possessing several aliphatic amino acids. Although the threonine and the aliphatic amino acids are not unique to the region of the protein surrounding Tyr20, there are a number in close proximity suggesting that this region of the protein adopts a less structured and more dynamic conformation within the S100A9 fibrils.

## 3. Materials and Methods

### 3.1. Expression and Purification of S100A9

S100A9 was expressed and purified as described before [22,23]. Briefly, wild-type S100A9 was expressed as inclusion bodies in *E. coli* BL21(DE3) using a pET expression system. All reagents were purchased from Sigma-Aldrich (Poole, UK). For unlabelled protein, 5 mL of overnight culture was used to inoculate 500 mL of 2YT media (200 rpm, 36 °C). The bacteria were grown to an OD_600_ of 0.8, and protein expression was induced through the addition of isopropyl-β-D-thiogalactopyranoside to a final concentration of 1 mM. After 4 h, the bacteria were harvested by centrifugation (9000× *g* for 20 min). Protein labelled with ^15^N and/or ^13^C was expressed in M9-labelled media using ^15^N-labelled ammonium chloride and/or ^13^C-labelled glucose as the sole nitrogen and carbon sources. The M9 media (50 mL) were inoculated with 0.5 mL of overnight culture, and these were grown to an OD_600_ of 0.8. This expanded culture was used to inoculate 450 mL of M9 media, and the bacteria were grown to an OD_600_ of 0.8 prior to induction of protein expression through the addition of isopropyl-β-D-thiogalactopyranoside to a final concentration of 1 mM. After 4 h, the bacteria were harvested via centrifugation (9000× *g* for 20 min).

Following expression, the bacteria were resuspended in 20 mM Tris buffer (pH 7.2) containing 1 mM EDTA. After treatment with lysozyme, the cells were disrupted via sonication (5 min, 50% duty cycle, 5 s on/5 s off). The insoluble material was subsequently pelleted via centrifugation (20,000× *g*, for 20 min). The resulting pellet was resuspended in 1 M urea to remove weakly bound materials from the inclusion bodies. The inclusion bodies were isolated by centrifugation (20,000× *g*, for 20 min) and subsequently dissolved in 6 M guanidium hydrochloride.

The S100A9 was refolded via dialysis three times against 4 L of buffer (20 mM Tris, pH 7.2, 1 mM dithiothreitol). Following clarification via centrifugation (20,000× *g* for 20 min), the refolded S100A9 was isolated via anion exchange chromatography (HiTrap Q, 5 mL, Cytiva, Chalfont, UK). The sample was loaded in 20 mM Tris buffer (pH 7.2) and eluted using a linear gradient of increasing concentration of 1M NaCl in 20 mM Tris buffer (pH 7.2). The fractions containing S100A9 were concentrated to 5 mL using a VivaSpin centrifugal filter (5000 MWCO). The S100A9 was then loaded onto a gel filtration column (HiLoad 16/600 Superdex 75pg, Cytiva, Chalfont, UK) pre-equilibrated with 25 mM ammonium bicarbonate, pH 7.2, at a flow rate of 1 mL min^−1^. The monomeric S100A9 was collected and lyophilized prior to storage at −20 °C.

### 3.2. Thioflavin-T Fluorescence Fibrilization Assays

Fibril formation was monitored via Thioflavin-T (ThT) fluorescence assay. Lyophilized S100A9 was dissolved in 20 mM Tris buffer (pH 7.2), and filtered through a 0.22 mm filter. Fibrilization assays were measured on a BMG Clariostar 96-well plate fluorimeter with excitation and emission filters set to 450 nm and 485 nm, respectively. Experiments were conducted with a final protein concentration of 250 mM in the presence of 25 mM ThT. The concentration of Ca^2+^ ions was adjusted through the addition of the appropriate concentration of CaCl_2_ dissolved in Tris buffer (pH 7.2). The experiments were conducted at 41 °C, with measurements taken every 5 min.

### 3.3. Circular Dichroism

Lyophilized S100A9 was dissolved in 20 mM Tris buffer (pH 7.2), with the appropriate Ca^2+^ ion concentration obtained through the addition of CaCl_2_. The resulting samples were filtered with a 0.22 mm filter. Synchrotron radiation CD measurements were recorded at B23 beamline, Diamond Light Source, UK, with access provided through awarded beamtimes (SM19915). Measurements were conducted on the module B beamline using a quartz 96-well plate with a path length of 0.02 cm. All measurements were conducted at 298 K. CD spectra were measured from 165 to 260 nm in 1 nm steps. All spectra are the result of 16 scan averaging. Quantitative analysis of the secondary structural contributions was performed using the Dichroweb Server [40], using the SELCON algorithm and Basis Set 7, which has been optimised for use with synchrotron radiations CD spectra [41,42].

### 3.4. Differential Scanning Fluorimetry (DSF)

Protein samples contained 100 μM S100A9, 20 mM Tris (pH 7.2), supplemented with 100 μM 1,8-anilinonaphthalene sulfonate (ANS) and different (0–20 mM) calcium concentrations (3 repeats for each sample, 20 μL for each repeat). DSF experiments were performed with a Rotor-Gene Q instrument (QIAGEN) using the blue channel (excitation 365 ± 20 nm, detection 460 ± 20 nm). Constant heating was applied at a rate of 1 °C/min from 25 °C to 99 °C. The data were normalized and analysed using MoltenProt software [43]. Thermal melting temperatures (T_m_) were determined using two equilibrium state model (temperature range for baseline estimation: 10).

### 3.5. NMR Spectroscopy

For liquid-state NMR spectroscopy, lyophilized S100A9 was resuspended in 20 mM bisTris buffer pH 7.2 and filtered through a 22 mm filter. Data were acquired over a range of calcium concentration through the addition of a concentrated CaCl_2_ solution. All NMR spectra were recorded at 298 K on a 600 MHz Varian Inova spectrometer (Yarnton, UK) equipped with 5 mm z-gradient triple-resonance cryoprobe. Gradient-enhanced HSQC ^1^H-^15^N spectra were recorded within Varian’s BioPack with acquisition times in the direct and indirect dimensions of 64 and 48 ms, respectively. Data were processed with solvent suppression and 15 Hz Gaussian broadening in both dimensions.

Solid-state NMR spectra were recorded on a 600 MHz DD2 Agilent NMR spectrometer (Yarnton, UK) equipped with 3.2 mm triple-resonance MAS probe. All data were acquired at 273 K (temperature set point), with 12.5 kHz MAS frequency. Homonuclear ^13^C/^13^C correlation spectra were acquired using adiabatic cross polarization from ^1^H to ^13^C using a 70 kHz ^13^C rf field and an experimentally optimised ^1^H field of ~82 kHz [44]. During both direct and indirect detection, SPINAL proton decoupling was applied with a field of 100 kHz [45]. During the mixing period, DAR recoupling was applied with a ^1^H rf field of 12.5 kHz [35,36,46]. All 2D data sets were acquired with States-TPPI in the indirect dimension and typically contained 192 complex data points [47]. All solution-state NMR data were processed in nmrPipe and analysed in using CCPN Analysis [48,49]. Solid-state NMR data were processed in matNMR v3.9 and analysed using home written scripts in Matlab v2022b [50].

## 4. Conclusions

In this study, we demonstrate that despite exhibiting a well-defined α-helical structure, when probed via circular dichroism, over the longer millisecond timescales probed via NMR, the S100A9 protein exhibits significant conformational plasticity, especially in the apo-state. By using ^1^H/^15^N HSQC spectra of ^15^N-labelled S100A9, we show that increasing concentration of Ca^2+^ limits this conformational plasticity, with the significant ordering of the protein structure occurring in two steps—the first at micromolar, and the second—at higher millimolar concentrations, consistent with the occupancy of the high and low-affinity Ca^2+^-binding sites. This reduction in conformation plasticity correlates with the reduction in the propensity of S100A9 to form fibrillar structures, as revealed via the ThT fluorescence assay. Despite the conformational plasticity that S100A9 exhibits in solution, even in the presence of a non-limiting concentration of Ca^2+^, S100A9 adopts a single well-defined fibrillar structure with an extensive rigid core and with smaller, more mobile regions of protein, presumably decorating the surface of the fibril.

## Data Availability

All data used in this manuscript will be made available through the University of Southampton’s electronic archive (e-prints: https://eprints.soton.ac.uk). DOI (to be completed upon acceptance).

## Supplementary Materials

The supporting information can be downloaded at: https://www.mdpi.com/article/10.3390/ijms241713200/s1.
