# Supplementary material for: The Stabilization of S100A9 Structure by Calcium Inhibits the Formation of Amyloid Fibrils
Source: Int J Mol Sci. 2023 Aug 25;24(17):13200. doi: 10.3390/ijms241713200 (PMC10488161; doi:10.3390/ijms241713200)
Supplement: Supplementary file 1 [file ijms-24-13200-s001.zip › ijms-2546307-supplementary.pdf]

Supplementary Material

The stabilization of S100A9 structure by calcium inhibits the formation of amyloid fibrils.

Ella Sanders[1], Rebecca Csondor[1], Darius Šulskis[2], Ieva Baronaitė[2], Vytautas Smirnovas[2], Luckshi Mahesewaran[1], Jack Horrocks[1], Rory Munro[1], Istvan Horvath[3], Ludmilla A. Morozova-Roche[3], Philip T.F. Williamson[1†]

[1]Centre for Biological Sciences, University of Southampton, Southampton, United Kingdom, SO17 1BJ.

[2]Sector of Amyloid Research, Institute of Biotechnology, Life Sciences Centre, Vilnius University, Lithuania.

[3]Department of Medical Biochemistry and Biophysics, Umeå University, Umeå, SE-90187, Sweden.

[†] *Corresponding author*

Supplementary Information.

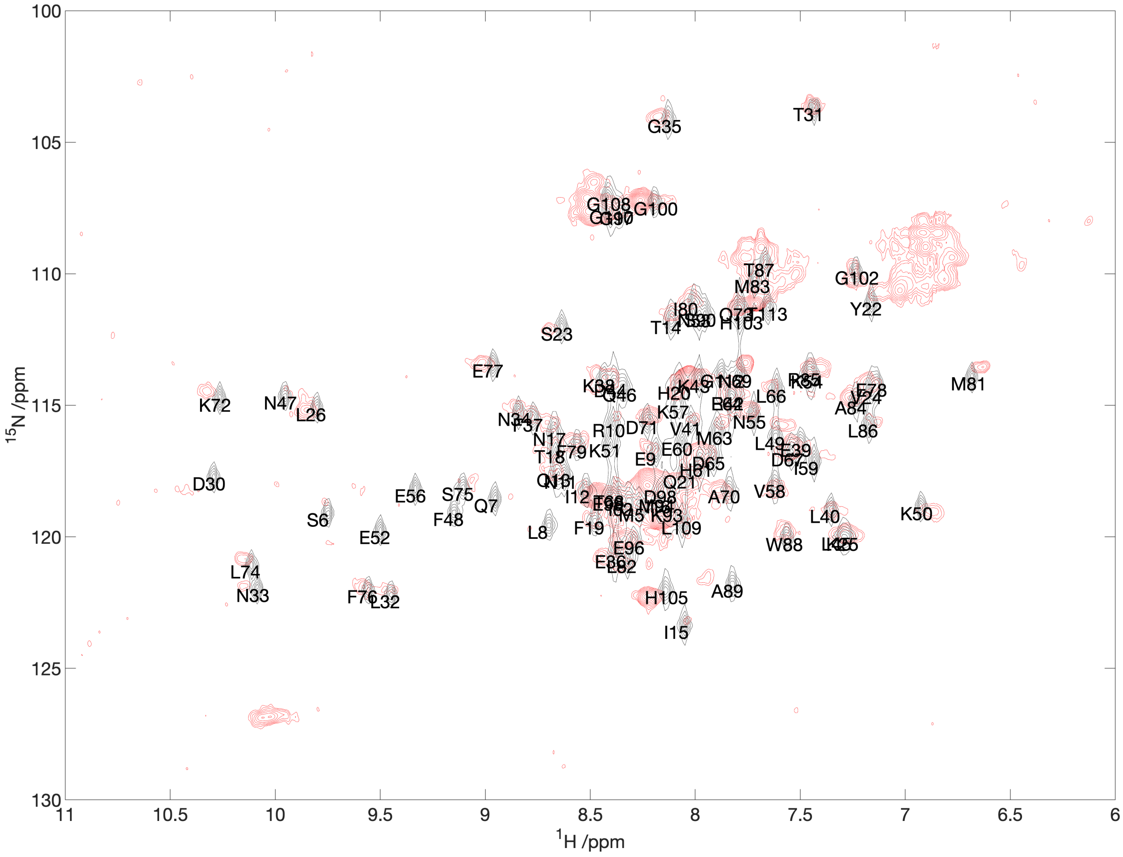

Supplementary Figure S1. ^{1}H/^{15}N HSQC spectra of S100A9 (100 μM in 20 mM bisTris buffer pH 7.2) with 4.0 mM $CaCl_2$ (red). Simulated ^{1}H/^{15}N HSQC spectrum of the backbone resonances of S100A9 based on the published resonance assignment of S100A9[43] (black).

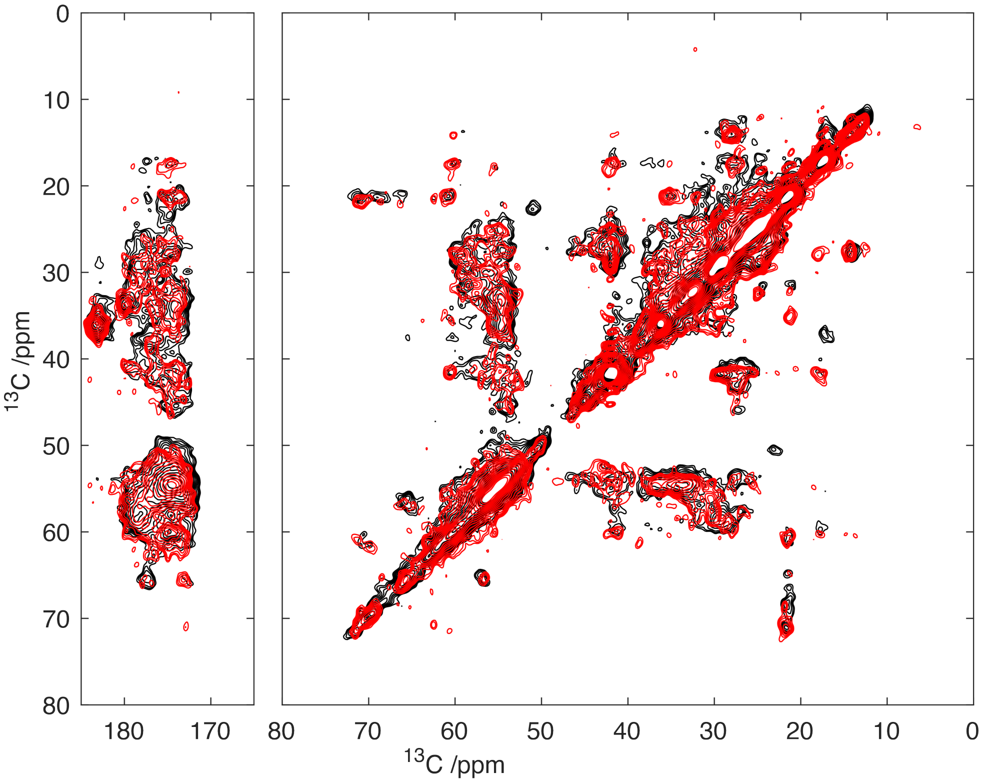

Supplementary Figure S2. 2D S100A9 fibril $^{13}C/^{13}C$ DARR spectra (black) overlaid with S100A9 microcrystal $^{13}C/^{13}C$ DARR spectra (red), both with a 25 ms mixing times, and processed with 100 Hz Gaussian broadening. Spectra were acquired at 600 MHz on a DD2 Agilent Spectrometer with 3.2mm MAS probe, spinning at 12.5 kHz. All data obtained at 0°C.
